# A Universal Stamping Method of Graphene Transfer for Conducting Flexible and Transparent Polymers

**DOI:** 10.1038/s41598-019-40408-w

**Published:** 2019-03-08

**Authors:** Bananakere Nanjegowda Chandrashekar, Ankanahalli Shankaregowda Smitha, Yingchun Wu, Nianduo Cai, Yunlong Li, Ziyu Huang, Weijun Wang, Run Shi, Jingwei Wang, Shiyuan Liu, S. Krishnaveni, Fei Wang, Chun Cheng

**Affiliations:** 1 Department of Materials Science and Engineering and Shenzhen Key Laboratory of Nanoimprint Technology, Southern University of Science and Technology, Shenzhen, 518055 P. R. China; 2 Department of Electronics, Yuvaraja’s College, University of Mysore, Mysuru, 570006 India; 3 Department of Electrical Engineering, Southern University of Science and Technology, Shenzhen, 518055 P. R. China; 4 Department of Physics and Center for 1D/2D Quantum Materials, The Hong Kong University of Science and Technology, Clear Water Bay, Kowloon, Hong Kong P. R. China; 5 Department of Studies in Physics, University of Mysore, Mysuru, India

## Abstract

Transfer method of chemically vapor deposition graphene is an appealing issue to realize its application as flexible and transparent electrodes. A universal stamping method to transfer as grown graphene from copper onto different flexible and transparent polymers (FTPs) reported here ensures simple, robust, rapid, clean and low-cost. This method relies on coating ethylene vinyl acetate (EVA) onto the as grown graphene, binding EVA coated graphene/Cu with FTPs and delamination by hydrogen bubbling process, which is analogous to the method used by stamping process where ink carries the imprint of the object onto any materials. The fate of the stamping method depends on how strongly the adhesion of EVA coated graphene/Cu with target FTPs. Interestingly, we have found that the thin film of EVA/graphene/Cu can only bind strongly with the FTPs of less than 25 µm in thickness and lower glass transition temperature value to the EVA while wide range of other FTPs are considered upon surface engineering to enhance the binding strength between FTPs and EVA. What’s more, the electrical performance was investigated with a demonstration of triboelectric nanogenerators which confirmed the reliability of graphene transfer onto the FTPs and prospect for the development of flexible and transparent electronics.

## Introduction

Since the discovery of mechanically exfoliated graphene^1^, production of high quality chemically vapor deposition (CVD) graphene for the industrial scale has remained challenging^2–4^. Large domain size graphene grown on Cu may explore the possibilities of realistic application upon transfer onto the dielectric substrates^5–9^. Graphene on polymer substrates are especially appealing as replacement of Indium tin oxide (ITO) and also one of the essential flexible and transparent electrodes for a wide range of optoelectronics devices such as touch screen displays and solar cells^10–12^. Polymethyl methacrylate (PMMA) polymer is used to transfer graphene from Cu metal substrate onto the dielectric substrates^13,14^, but only limited to study for the fundamental properties. Till date, as grown CVD graphene was transferred onto many different types of flexible polymers^15–21^. In majority of transfer case, PMMA and thermal releasing polymers are used as intermediate polymer to transfer graphene onto different polymers which scarifies the Cu substrates and induces cracks^19,22^. Roll to roll transfer of graphene onto polyethylene ptherapthalate (PET) was also achieved via thermal release tape^10^, epoxy resin^15^ and ethylene vinyl acetate(EVA)^23^ as binding source between graphene and PET. Roll to roll graphene transferred onto the EVA/PET by bubbling method^24^ and green transfer^23^ is hopeful to achieve the low cost, light weight, flexible and transparent electrodes. To our best of knowledge there is no report found on graphene transfer onto many other polymers, except PET, using EVA as binding agent for the industrial scale. EVA is explored against PMMA mediated graphene transfer for conformal contact with the target substrates^25,26^. Graphene transfer onto different polymers of characteristics properties suits wider range of applications still remains challenging and not many efforts have been made in this direction^27^. Recent advances in flexible electronics expect to soon realize the industrial production of graphene based hybrid transparent electrodes^28^. Our study met the challenge to transfer graphene onto different polymers by a fast and clean process.

**Figure 1:**
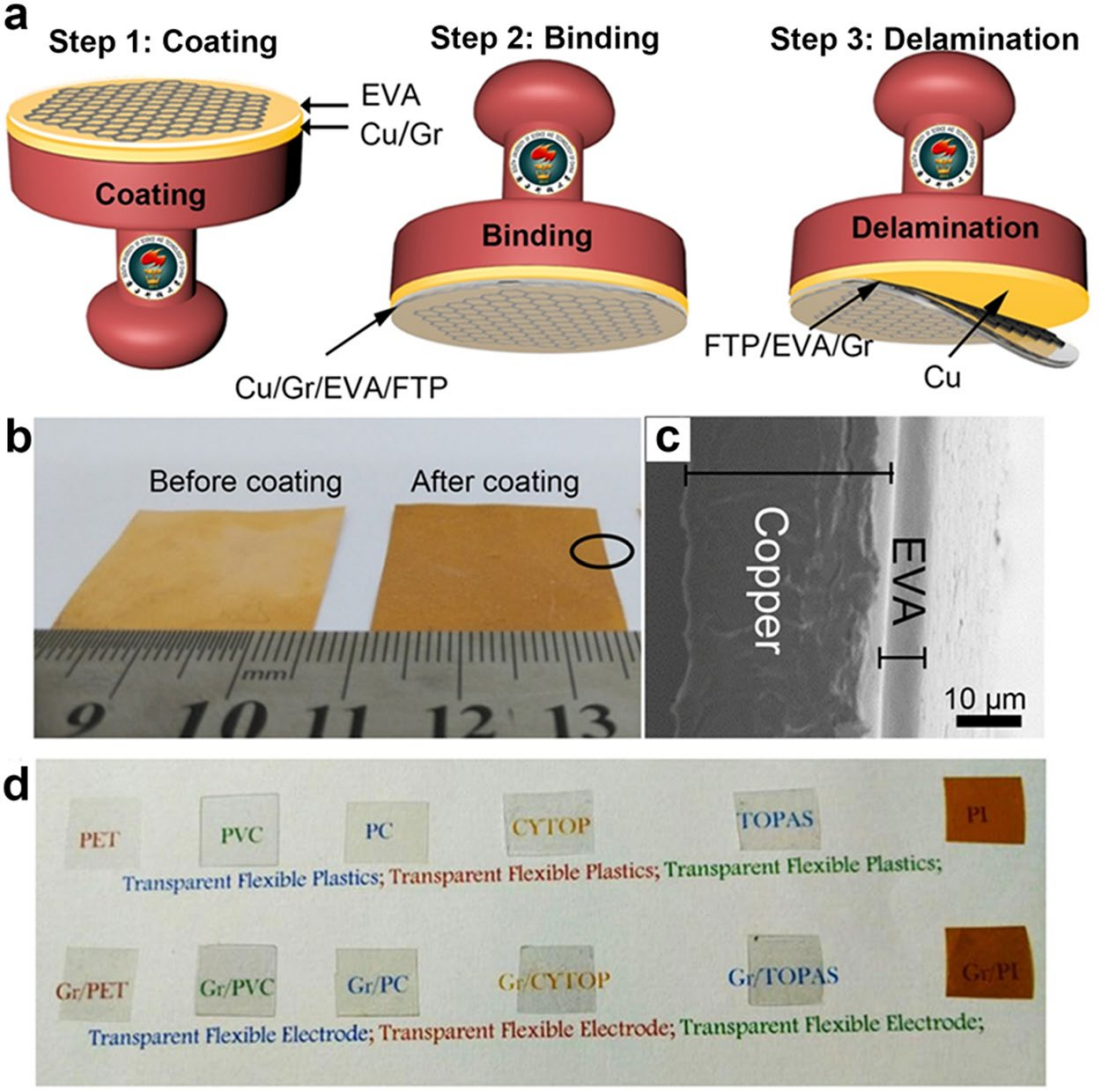
Schematic illustration of stamping method of graphene transfer: (**a**) Schematic illustration showing stamping method comprises three steps; (i) EVA coating on to as grown graphene/Cu, (ii) binding EVA/graphene/Cu onto FTPs and (iii) delamination of graphene/EVA/FTPs from Cu. (**b**) Photographic images of graphene/Cu before EVA coating (left) and graphene/Cu after EVA coating (right); the black circle indicate sampling position for data in c. (**c**) Vertical crossectional SEM image of EVA coated graphene/Cu. (**d**) Top, photograph of the different flexible and transparent polymers. Bottom, graphene after transferred onto different FTPs by stamping process.

In this work, we designed a stamping method to transfer as grown graphene on Cu onto different flexible and transparent polymers (FTPs) which is applicable to the large scale graphene requirement as flexible and transparent electrode in modern electronics. This innovative method (Figs 1a and S1) relies on coating EVA onto the as grown graphene on Cu, binding EVA coated graphene/Cu with different polymers and delamination by hydrogen bubbling process, which is analogous to the method used by stamping process where ink carries the imprint of the object onto any materials. EVA has characteristic properties such as excellent transparency, flexibility and adhesivity as a function of temperature, which is already being used as encapsulating layers in solar panels^29^, is the major role in this transfer process. Next generation of wearable/bendable electronics demands the potential supply of flexible and transparent polymers as transparent electrode substrates in the areas of energy conversion, environmental monitoring, healthcare and communication and wireless network^30^. Although either big or small differences in polymer properties such as transmittance, flexibility, oxygen and water permeability, temperature resistance may largely impact on the functional properties desired for the respective applications. We chose thermoplastic transparent and flexible polymers, but not limited to, such as polyethylene terephthalate (PET), polyimide(PI), polycarbonate(PC), polyvinyl chloride(PVC), TOPAS (thermoplastic polymer mr-I T85) and CYTOP (an amorphous fluoropolymer type: CTL-809M, was purchased from AGC Asahi Glass) as target substrates for graphene based conductive film which could open the new avenue either in the modern electronics. The fate of the stamping method depends on how strongly the adhesion of EVA coated graphene/Cu with target FTPs. Interestingly, we have found that the thin film of EVA coated on graphene/Cu can only bind strongly with the FTPs of less than 25 µm in thickness and lower glass transition temperature (Tg) value to the EVA without any pretreatment (unmodified-FTPs) while wide range of other FTPs such as higher or lower Tg to that of EVA with low thermal conductivity in thicker polymer substrates are considered upon surface engineering (surface engineered–FTPs) to enhance the binding strength between FTPs and EVA. What’s more, the electrical performance was investigated with triboelectric nanogenerators (TENG)^31–34^, which confirms that our transfer process is reliable for the different polymers and prospect for the development of flexible and transparent electronics.

## Methods

### CVD graphene growth

A commercially available Cu foil (98% purity, 25 μm thick, Alfa Aesar #46565) was electrochemically polished in electrolyte solution composed of phosphoric acid and ethylene glycol(V/V = 3:1) with a bias of 2 V for 30 minutes. The electropolished Cu foils was washed thoroughly in alcohol and water sequentially to make sure the Cu surface was free from electrolytic residues. A monolayer graphene was grown on electropolished Cu in a low-pressure CVD system. Cu was loaded into the 4 inches diameter long tubular quartz at hot center of the furnace and degasses the furnace for the initial pressure of 7 × 10^−2^ torr. Cu was allowed to be annealed for 60 minutes at 950 °C with a passage of 50 sccm of H_2_ gas maintain a tube pressure of 1 torr. Then 20 sccm methane, corresponding to a pressure of 2 torr, was introduced for 30 minutes to grow the continuous monolayer graphene.

### Graphene transfer by stamping method

After CVD grown graphene on copper, an EVA solution (1–4 wt% dissolved in cyclohexane, Aladdin Industrial Corporation, Shanghai) was coated onto the graphene side of the copper by spin/spray/blade coating process. Prior to coating process, Cu was flattened using glass rod; edges of the graphene/Cu were covered with scotch tape to avoid the coating of EVA onto the backside of the graphene/Cu. Spin coating is done with two spinning steps; 200 rpm for 30 seconds and 400 rpm for 60 seconds. Two times coating was done to ensure the continuity of the thin film EVA. In spray coating process, EVA solution was kept at hot container to make solution free flow through the nozzle. The spray nozzle movement and pressure were adjusted to 20 mm/s and 0.2 Mpa, respectively. We also demonstrated blade coating process, which is most suitable for the large scale graphene transfer attributed to roll to roll process. Speed of the blade movement was adjusted to 10 mm/s while distance between the blade and sample to be coated was set to 0.5 mm. The temperature of the sample holder both in spray and blade coating were kept at 50 °C to make fast evaporation of the solvent. After complete evaporation of solvent after being kept in dry box for 60 minutes, the resultant EVA/graphene/Cu was bound onto any of the flexible polymers (cleaned using isopropanol and blow-dried with a nitrogen gun) using hot lamination method to form FTP/EVA/graphene/Cu. Finally, graphene/EVA/FTPs is delaminated from the metal substrate by electrochemical hydrogen bubbling method^21,33^ and dried in air.

### Characterization

The morphology was characterized by optical microscopy (Olympus BX51), scanning electron microscopy (ZEISS-Merlin) and atomic force microscopy (Bruker). Quality of the CVD grown graphene transferred onto Si/SiO_2_ was evaluated using a Raman spectroscope (Horiba, LabRAM HR Evolution) with a laser excitation wavelength of 532 nm. Monochromatic Al X-ray (Physical Electronics 56000 multitechnique system) was used to analyze any metal residue remained after graphene being transferred onto FTPs. The transmittance measurement was examined using UV-Vis-NIR spectrophotometer (Perkin Elmer, Lambda 750 s, 190–3300 nm). Contact angle measurement was done by contact angle tester (AST VCA Optima XE). Sheet resistance measurement was carried out using four probe system (Guangzhou 4-probe Tech Co. Ltd., RTS-4) with probe spacings of ~1 mm.

### Fabrication of triboelectric nanogenerator

The final product from this transfer process graphene/EVA/FTPs film was used as the electrode of TENG. The fabricated device consists of a graphene/EVA/FTP electrode coated with CYTOP film (triboelectric layer) (18 × 20 mm) and a PET substrate coated with ITO films. Both two components were placed in an acrylic glass as a proof mass which can be driven by external vibration. The acrylic mass and transparent substrate were assembled in the elastic holder to make an arched structure with a dimension of 45 × 45 × 10 mm. The CYTOP polymer coated on the bottom plate is charged in a custom-built corona charging setup. The setup consists of a grounded electrode, a metal mesh grid (V_g_ = −2000 V) and a high-voltage probe tip (V_H_ = −6 kV). The device is driven by a mechanical shaker with controlled frequency and amplitude, where an accelerometer is used to monitor the acceleration during the measurement. The shaker is driven by an excitation signal generated from a signal generator (Brüel&Kjær, LAN-XI 3160) and a power amplifier (Brüel&Kjær, 2719).

## Results

Monolayer graphene is grown on electrochemically polished 25 µm thick copper foils by low-pressure chemical vapor deposition (LPCVD)^2^. Both optical microscopy (OM) (Fig. S2a) and scanning electron microscope (SEM) (Fig. S2b) of graphene transferred onto SiO_2_ by PMMA revealed continuous monolayer graphene with bilayer or multilayer graphene domains. Figure S2c shows the Raman spectroscopy of the graphene on SiO_2_ confirms the high quality graphene by showing the negligible D peak which is raised commonly due to the PMMA mediated transfer. Figure 1a shows the schematic of stamping method to transfer CVD-grown graphene from Cu substrate to a FTPs comprises three major steps; (i) EVA is coated onto the as grown graphene on Cu by any of the three modes such as spin coating for small sample, spray coating and blade coating for large samples (Fig. S1a). The coated sample represents as ethylene vinyl acetate/graphene/copper (EVA/graphene/Cu) thin film. Figure 1b shows the photograph which distinguishes the graphene on Cu before and after EVA coating, where bright contrast of Cu/graphene becomes dull upon EVA coating which revealed the EVA film formation. A thin close contact of EVA upon blade coating is evidenced with the vertical cross-sectional SEM (Fig. 1c). (ii) Binding the EVA/graphene/Cu thin film with the target FTP substrate using hot lamination (Fig. S1b) to form FTP/EVA/graphene/Cu stack as similar to the graphene transferred onto commercially available EVA/PET^23,24^. Hot lamination in our graphene transfer method is reliable for the flexible polymers as coated EVA layer on Cu/graphene unchanged the flexible property. Roller temperature was adjusted according to the Tg of EVA. Higher temperature than Tg of EVA makes the FTPs film deform. We found some interesting mechanisms of binding EVA coated graphene/Cu thin film with the smooth surface morphology FTPs (less than 25 µm) having similar Tg of EVA such as TOPAS, CYTOP, PET. 25 µm thin FTPs with lower Tg undergoes easy deformation at 120 °C, since as grown graphene on Cu has considerable roughness^35^, smoother FTPs adhered tighter with EVA/graphene/Cu due to roughness impression carried from graphene/Cu. But in the case of thicker FTPs of 100 µm such as PET, PC, PVC, we cannot found tight adhesion with EVA/graphene/Cu due to insufficient supply of required temperature to attain Tg value for thicker FTPs in lamination process. (iii) Delamination of FTP/EVA/graphene/Cu stacks to transfer graphene onto FTP/EVA from Cu substrate using electrochemical hydrogen bubble method by polarizing Cu/graphene/FTP at 2 V^24,36,37^ (Fig. S1c). Figure 1d shows the photographs of the graphene/EVA/FTP (bottom row) which are comparable to that of EVA/FTP (top row), indicating that transfer process has little effect on the transmittance of target FTP upon graphene transfer. Note that all FTPs are resistant to the NaOH (electrolytic solution) and the method ensures successful graphene transfer without damaging either Cu substrates or EVA layer coated on graphene/Cu. Since FTP/EVA/graphene/Cu stack was used as cathode in the delamination process, H_2_ bubble generates between graphene and the Cu substrates which leads to the delamination of graphene onto the EVA/FTPs. Oxidation of graphene/EVA/FTPs is avoided while Cu undergoes slight oxidation due to the basic electrolytic solution but it favors the growth of high quality graphene for the second time^23^. The graphene/FTPs film is rinsed with deionized water and blow dried with nitrogen to ensure it to be free of chemical residues.

Prior to the coating EVA solution onto the graphene on Cu, the method further comprises the step of flattening the CVD-grown graphene on Cu foil (Fig. S3a). Spin coating is popular and commonly used to transfer graphene using PMMA solution because it helps to control the thickness of the thin film formation of PMMA layer^13^. The innovation of our work relies on the thin film deposition of EVA onto as grown graphene on Cu and surface engineering of FTPs, where EVA acts as a binding agent between graphene and target substrates. In the process of coating, EVA solution preparation and optimization process is very important because we are the first using EVA to transfer graphene on to different FTPs. The preparation method is as follows: 1–4 wt.% EVA solution is made by dissolving EVA and stirring in cyclohexane at 75 °C. In fact, we demonstrated the coating method based on the sample size; spin coating is generally suitable for small size graphene since it is easier and makes the process fast. A thin layer of 1% EVA was spin coated twice to ensure a continuous layer, parameters were set similar to the PMMA coating^38^. On the other hand, for large area of graphene samples especially for roll to roll CVD graphene, either spray coating or blade coating process enables the process efficient and easier. Since the diameter of spray nozzle is small, 1% EVA is used in the spray coating method to avoid the blockage of nozzle. It should be noted that spray coating can control the tight contact of EVA with graphene upon adjusting the spray pressure to form the compact thin uniform EVA film. Multi spray coating deposition can also be done to control the thickness, which is attributed to achieve graphene transfer onto surface engineered polymers. To obtain thicker film on large area graphene, 4% EVA was coated by blade coating machine. By adjusting the distance between Cu/graphene and blade, thickness can be controlled up to 20 µm. Detailed procedure of coating method is described in experimental part. The samples after deposition were kept at room temperature for 60 minutes to evaporate the solvent. By comparing the vertical cross-sectional SEM images of Cu/graphene (Fig. S3d) and EVA coated Cu/graphene (Fig. S3e), the continuity of EVA layer without any disruption was confirmed. Note that there is no reaction found between EVA solution and graphene/Cu metal substrate, though it helps the formation of thin film very fast due to fast evaporation of the solvent. Both blade coating and spray coating can be integrated in the roll-to-roll processing of graphene transfer.

## Discussion

**Figure 2:**
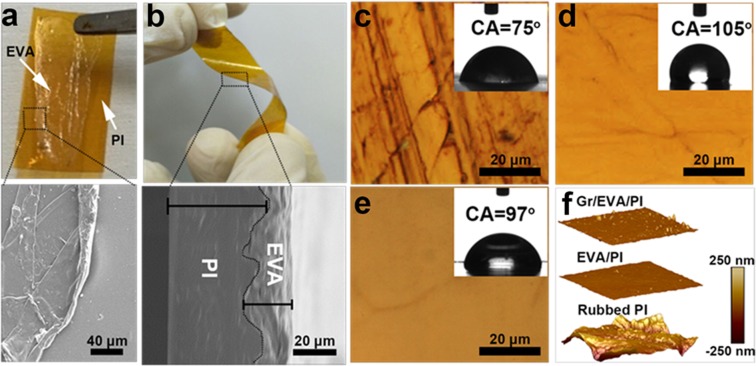
Surface engineering of FTPs: Photograph of PI/EVA/graphene before surface engineering (**a**) and after surface engineering (**b**); dotted square in (**a**) shows that the EVA/graphene adhesivity on PI is very weak, where dotted square (**b**) is spotted for vertical cross-sectional SEM image revealing tight adhesion of EVA with FTPs. (**c**) OM image of PI rubbed with sand paper, EVA coated on rubbed PI (**d**) and EVA/graphene transferred onto the EVA coated PI (**e**), where inset shows the contact angle measurement of the respective samples. (**f**) AFM image to show the surface morphology of rubbed PI (bottom), EVA coated PI (middle), and graphene transferred onto the EVA coated PI (top).

Interestingly, we found our EVA mediated stamping method of graphene transfer onto smooth FTPs can only be realized when their thickness and Tg is less than 25 µm and ~140 °C, respectively. Weak binding force between the two smooth surface polymers films were noticed in the fabrication of graphene/metal nanowire transparent electrodes^39^, which signalling that the surface engineering of the target FTPs are deciding factors to clamp thin film EVA to achieve efficient graphene transfer in our stamping method. It is also reported that EVA film shows excellent adhesive bonding to solar glass which are rough in surface^40^. In addition, our method can also extend to FTPs thicker than 25 µm or with higher Tg temperature than EVA after proper surface engineering. The basic prerequisite of the target FTPs substrate should be surface roughness and hydrophobicity. Surface engineering in our method comprises two steps; (i) FTPs are and blasted to make the surface rough with the formation of crest and trough in large scale. (ii) Fill the crest and trough by coating 1% EVA solution using various methods. Generally, graphene transfer is only valid onto smooth surface materials for electronic applications^41^. To transfer graphene onto a wide variety of FTPs having higher Tg value to that of EVA and low thermal conductivity in thicker polymer substrates, surface engineering is inevitable. EVA mediated transfer overcome the challenge to transfer graphene onto rough surface by providing smooth surface basement to graphene upon EVA coating onto the target rough surface polymers. Here, we used but not limited to PI as FTP target substrates to demonstrate graphene transfer onto the smooth surface with a higher Tg value to that of EVA. Graphene transfer from Cu/graphene/EVA stack onto the PI is unsuccessful, where arrow in the Fig. 2a shows the EVA/graphene detachment from PI substrate. EVA/graphene thin film detachment from ultra-smooth PI substrate is clearly seen in SEM image (square mark of Fig. 2a), which signs that the graphene transfer can only be done upon surface engineering of target polymer substrates of higher Tg value and higher thickness (Fig. 2b).

Figure S4a shows the schematic illustration of FTPs surface modification, surface was thoroughly rubbed by sand paper, forming uneven surface with crest and trough and the same was made smooth by filling EVA solution by spin coating method. Crest and trough of rubbed FTPs were clamped tightly by the coated EVA which forms the flat surface, as shown in the vertical cross-sectional image of SEM (square mark of Fig. 2b). Further surface morphology characterizations of surface engineered-FTPs and graphene transfer onto the FTPs were carried out by OM and atomic force microscopy (AFM). OM shows that the crest and trough in FTPs ensures that the entire surface is uneven (Fig. 2c), while it turned to flat uniform surface upon EVA coating (Fig. 2d) and finally graphene transferred onto the EVA coated FTPs remains surface flat which is identified with the graphene grain boundary (arrow in Fig. 2e). Our AFM observations show that the FTPs of higher Tg rubbed with sandpaper bears the uneven surface (Fig. 2f; bottom) with random crest and trough of several micrometers (line section of the surface morphology; Fig. S5a), which helps to clamp the EVA very tight (Fig. 2b). Upon EVA coating, crest and trough were filled to form a thin uniform film (Fig. 2f; middle) to satisfy the surface requirement to transfer graphene, surface roughness was decreased to several nanometers (Fig. S5b). After graphene transferred onto the surface engineered-FTPs, surface roughness was found to be quite increased as compared to the EVA coated FTPs before transfer, which is due to the surface morphology of graphene grown on copper^16^ (Fig. 2f; top and Fig. S5c). Contact angle (CA) measurement was carried out to measure to the surface behavior of surface engineered FTPs before and after graphene transfer. In fact, rough surface FTPs shows hydrophilicity due to the rough surface morphology (CA = 75°, Fig. 2c), while EVA coated FTPs before (CA = 105°, Fig. 2c) and after Gr transfer (CA = 97°, Fig. 2e) remains hydrophobic which strongly shows that the graphene transfer upon surface engineered polymer is successful by stamping method. In contrast, topographical AFM images shown in Fig. 2f distinguishes the surface roughness of FTPs rubbed with sand paper (bottom), smoother upon EVA coating (middle) and finally turns to be negligible rough after graphene transfer (top).

**Figure 3:**
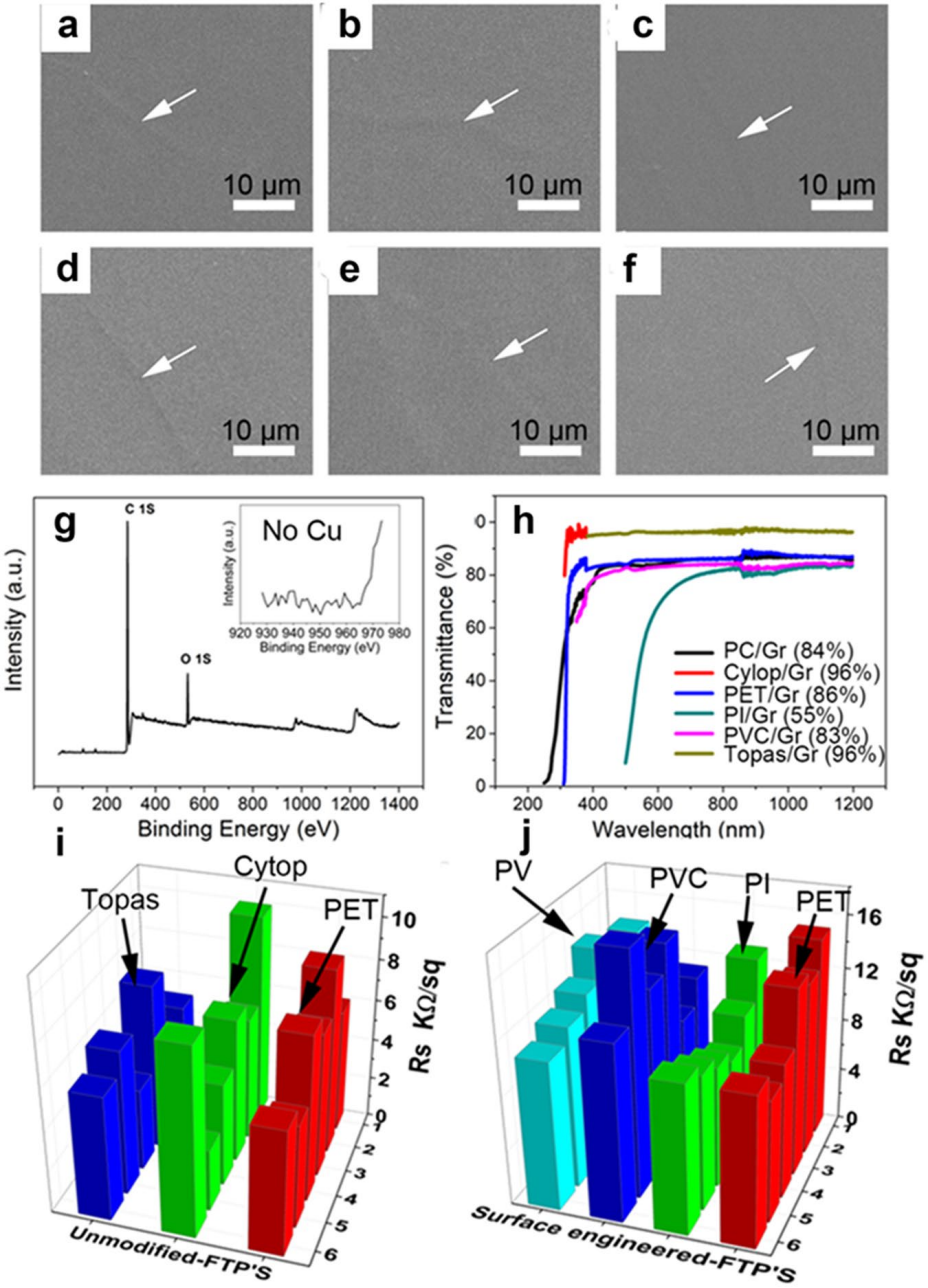
Characterization of graphene transfer by stamping method: SEM images of graphene/EVA on unmodified FTPs such topas (**a**), cytop (**b**) and PET (**c**). On the other hand graphene transferred onto EVA coated surface engineered-FTPs such as PVC (**d**), PC (**e**) and PI (**f**). (**g**) XPS spectra of EVA/graphene on PI. Inset, deconvoluted XPS spectra shows no Cu peaks. (**h**) UV-visible spectra of graphene/EVA on different FTPs. Sheet resistance map of graphene/EVA on unmodified FTPs (**i**) and EVA coated surface engineered-FTPs (**j**).

It is noteworthy that graphene grown on Cu foil by CVD shows high quality (Fig. S2). Surface morphology of the graphene on different polymers by stamping method was characterized by SEM. The graphene grain boundaries (arrow mark in Fig. 3a–f) are observed as the characteristics confirmation of graphene transfer onto the different polymers such as TOPAS, CYTOP, PET, PVC, PC and PI. No voids or cracks are seen from our transfer, which confirms the good contact between the target substrates and the EVA/graphene transferred from the Cu substrates. Surface of graphene/EVA on polymers shows slight wavy morphology due to the surface engineered FTPs substrates and the graphene/Cu. In the hot lamination process, EVA softens at 120 °C and mimics the surface morphology of both the FTPs and Cu, which results in the rough graphene/EVA/FTPs surface compared to that of EVA/FTPs before graphene transfer, as shown in the AFM image of Fig. 2f (See Supplementary Fig. S5c). However, OM image of graphene/EVA/FTP shows the confirmation of graphene transfer in large area, where graphene grains on Cu can be compared with the graphene grains on EVA/FTPs (Fig. S6a). Following coating and binding process, electrochemical bubbling method results the final graphene on FTPs; we found no surface contamination on graphene/EVA/FTPs by XPS and UV-Visible spectra. Figure 3g shows the XPS full spectra of graphene/EVA/FTPs. Two predominant XPS peaks of C 1 s and O 2 s found at 284.5 eV and 530 eV, which are ascribed to the sp2 carbon of graphene and the oxygen in EVA, respectively. The deconvoluted XPS spectra of graphene/EVA/FTPs in inset of Fig. 3g shows no predominant Cu peak in the binding energy between 930 and 960 eV at the detection limit of XPS. This observation indicates that the coating-lamination-bubbling process in the stamping method is efficient to transfer graphene/EVA from Cu onto FTPs. We then evaluated the UV-Visible spectrum of graphene/EVA/FTPs to confirm whether the quality of transparency is affected in the transfer process. Fig. S6b shows the UV-Visible spectrum of graphene transferred onto EVA/FTP showing 97.4% transmittance close to the theoretical value of graphene/Quartz, which confirms that our transfer process is successful with no surface contamination in the entire transfer process; note that the substrate transmittance was subtracted. The transmittance of graphene/EVA on different FTPs such as TOPAS, CYTOP, PET, PC, PVC and PI were evaluated showing that original transmittances of the FTPs were unaffected upon graphene/EVA transfer. The total T% values were found for graphene on different FTPs such as TOPAS, CYTOP, PET, PC, PVC and PI are 96%, 96%, 86%, 84%, 83% and 55%, respectively (Fig. 3h). Conductivity is one of the main concerns of graphene transferred onto FTPs. Sheet resistance of graphene/EVA/FTPs was evaluated with four point probe system. The large area of graphene on different-FTPs was measured with sheet resistance and the result value lies between 1–10 kohm/sq, which is acceptable range of graphene on EVA/PET by green transfer^23^. Because of high sheet resistance of polycrystalline graphene on dielectric substrates, metal nanowires are used to fabricate graphene based hybrid transparent electrodes to perform equivalent to that of ITO electrodes^24,42^. Figure S6c shows the schematic illustration for the reason of higher Rs found at graphene/EVA/FTPs. Four probes shown in the Fig. S6c spotted at within the grain size or between the large grain size Rs value is considerable lower than that of four probes spotted at small grains or higher grain boundary region results in higher Rs of graphene/EVA/FTPs. Either with the improved growth of large area domain size graphene on Cu^43^, metal nanowire networks^44^ or chemical doping^45^ are strong strategic direction that our transfer method is efficient to resolve the higher Rs value in graphene/FTPs. In contrast, graphene on unmodified-FTPs shows a Rs of 10 Kohm (Fig. 3i) while graphene on surface engineered-FTPs shows higher Rs of about 10–20 K Ohm (Fig. 3j), indicating the surface engineering process affects the electrical properties slightly. The distribution curve of sheet resistance of graphene on different unmodified FTPs is observed over the 3 × 4 cm graphene/EVA/FTPs is found to be 1–10 k ohm/sq, which are measured with the typical probe spacing 1 mm (Fig. S6d).

**Figure 4:**
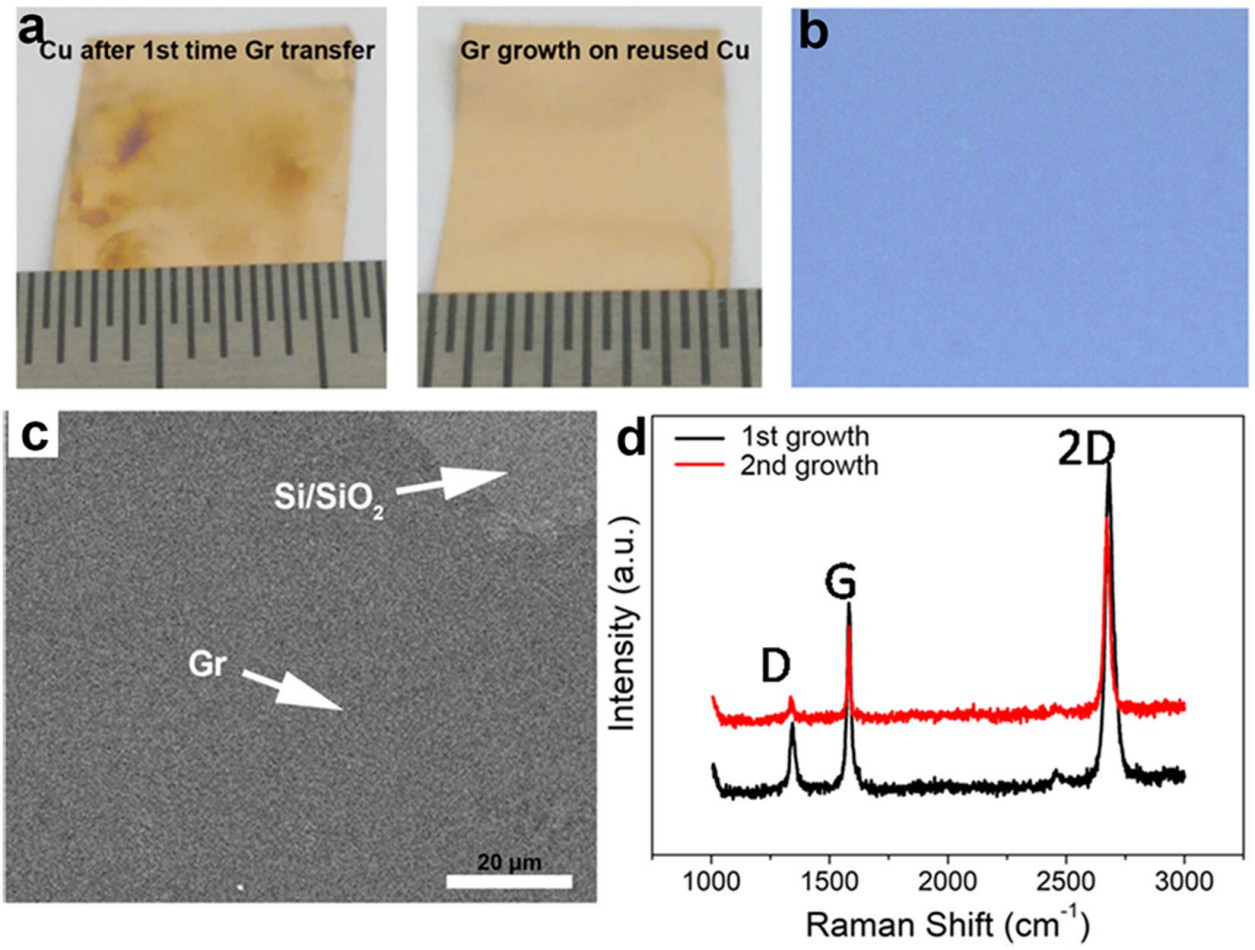
Repeated graphene growth on reused copper: (**a**) Left, photograph of Cu after the first time graphene growth and graphene/EVA thin film transfer by stamping method. Right, graphene grown on Cu foil after first time graphene/EVA transfer. (**b**) OM and (**c**) SEM image of graphene transferred onto SiO_2_ by PMMA transfer method; arrow represents the contrast of SiO_2_/Si where it distinguishes the contrast of graphene. (**d**) Raman spectra of graphene transferred onto Si/SiO_2_ where black line and red line corresponds to the first time graphene grown on Cu and second time graphene grown on reused Cu, respectively.

The Cu foil was not damaged by any process involved in stamping method (Fig. 1b), neither of chemical residue remained while EVA coating on graphene/Cu or tore Cu foil in the lamination and bubbling method. Vertical SEM images of Cu after graphene/EVA transferred onto FTPs show that there are no such mechanical damages found on Cu substrates (Fig. S3f). Figure 4a (left) shows the Cu foil after graphene/EVA thin film transferred onto the FTPs showing some part undergoes oxidation which is an advantage for the growth of high quality graphene^46^, while graphene grown upon reused Cu (right of Fig. 4a) turns to be shine which is similar to the Cu used to grow graphene at first time (Fig. 1b; left). The high-quality nature of graphene on reused Cu is evidenced by OM (Fig. 4b), SEM (Fig. 4c) and Raman spectrum (Fig. 4d). The enlarged domain size and less grain boundary may favor the quality improvement of graphene on FTPs, which is significant for economic industrial scale.

**Figure 5:**
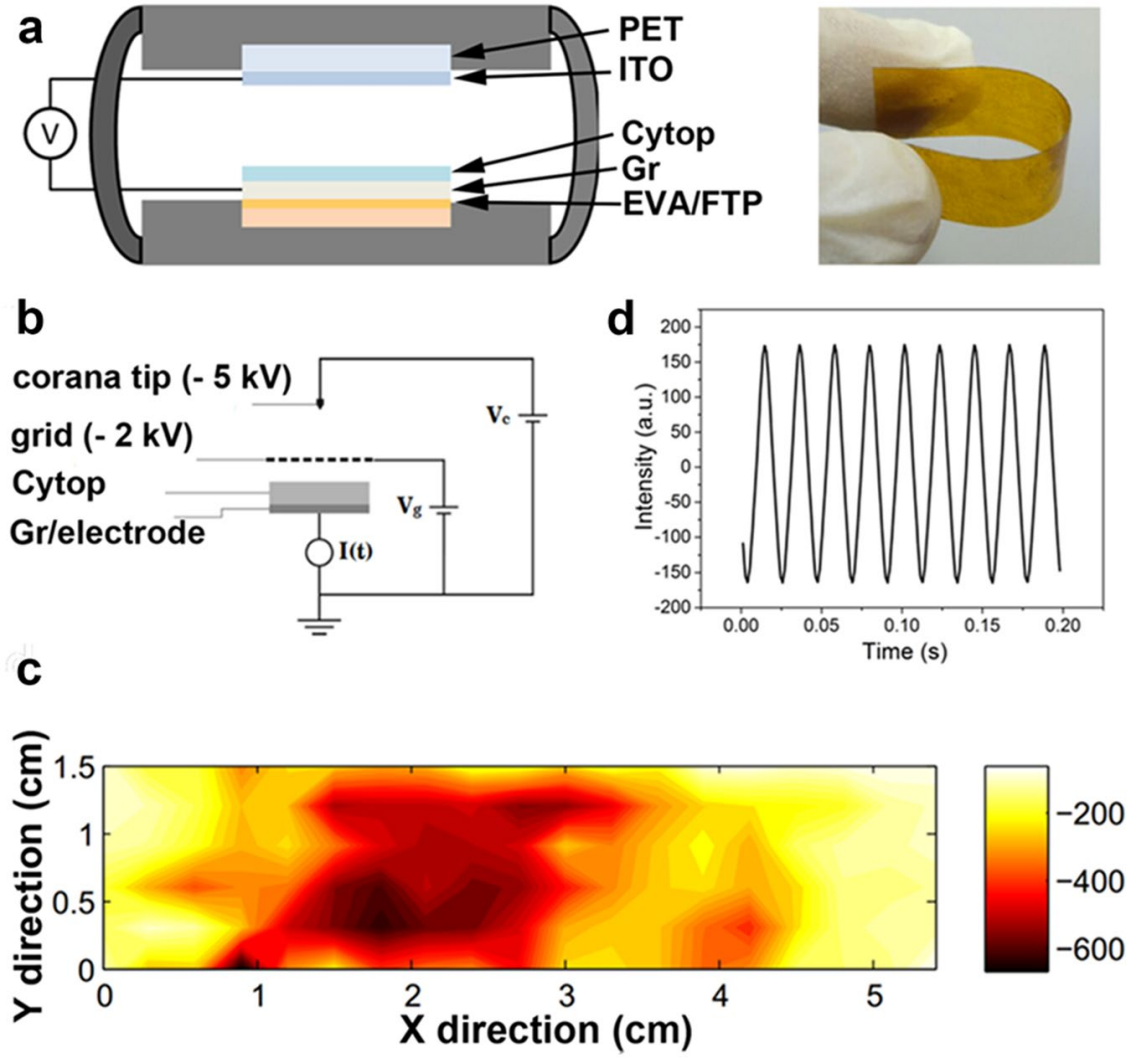
Fabrication of triboelectric nanogenerator: (**a**) Schematic diagram of graphene based flexible and transparent triboelectric device. Right, photograph of graphene/EVA/FTPs used in triboelectric nanogenerator showing high degree of flexibility. (**b**) Schematic diagram of a corona charging setting, (**c**) distribution plot of surface charge after corona charging. (**d**) A vibration plot of graphene/EVA/FTPs based triboelectric device at the vibration source of 45 Hz.

**Figure 6:**
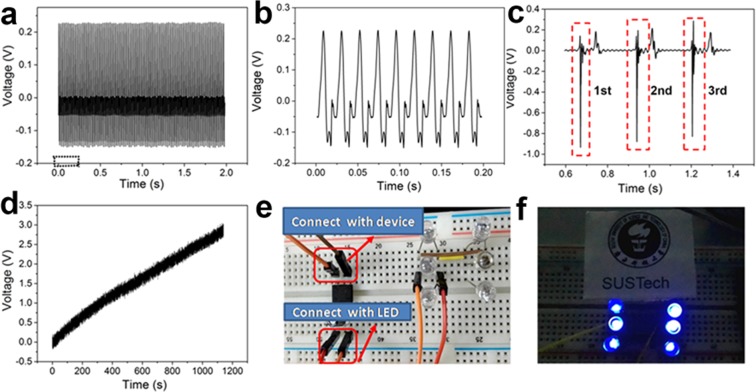
Electrical characterizations of graphene/EVA/FTPs based triboelectric nanogenerator: (**a**) Output voltage for device based on FTPs/EVA/graphene/cytop VS ITO/PET. Note that graphene here is used as back electrode. (**b**) Enlarged form from the red box in (a). (**c**) Hand Driven out put voltage from FTPs/EVA/graphene/cytop VS ITO/PET. Each contact output performances were shown in red dotted box. (**d**) Charging Curve for 10 μF capacitor based on FTPs/EVA/graphene/cytop VS ITO/PET. (**e**) Photograph of the circuit map to power LED. (**f**) LED’s lightened by 10 μF capacitor after being charged.

To confirm the graphene transfer performance on the electrical conductivity and flexibility, we have applied graphene/EVA/FTPs electrodes in the TENG. The fabricated device consists of a FTPs/EVA/graphene electrode coated with CYTOP film (18 × 20 mm2) and a PET substrate coated with ITO films sketched in Fig. 5a. The ITO plays dual roles as electrode and contact surface, while CYTOP plays the role as the other contact surface. Graphene is used as back electrode. Figure 5a (right) shows the photograph of the graphene/EVA/PI revealing the bending capability without causing any damage to graphene/EVA. To fabricate the device, cast acrylic glass was prepared as a proof mass which can be driven by external vibration. Both the acrylic mass and transparent substrate were assembled in the elastic holder to make an arched structure with a dimension of 45 × 45 × 10 mm^3^. The CYTOP polymer coated on the bottom plate is charged in a custom-built corona charging setup (Fig. 5b). The setup consists of a grounded electrode, a metal mesh grid (V_g_ = −2000 V) and a high-voltage probe tip (V_H_ = −5 kV). After charging for 15 min, the surface potential of the electrets layer is mapped in Fig. 5d. The energy harvesting performance of the device is characterized with a shaker setup shown in Fig. S7a. Our device is driven by a mechanical shaker with controlled frequency and amplitude, where an accelerometer is used to monitor the acceleration during the measurement. The shaker is driven by an excitation signal generated from a signal generator (Brüel&Kjær, LAN-XI 3160) and a power amplifier (Brüel&Kjær, 2719). Figure 5c shows a typical driven vibration with amplitude of 170 m/s^2^ at 46 Hz. To find the optimal work frequency, we have explored the relationship between the output power of the TENG and the frequency of the vibration source under different amplitudes, as shown in Fig. S7b. An output power peak can be seen at 46 Hz, which is exactly the same as the resonant frequency of the device. At resonance, the output voltage of TENG based on FTPs/EVA/graphene/CYTOP VS ITO/PET was presented in Fig. 6a with a closed-up view shown in Fig. 6b. The voltage peak reaches 0.23 V. Output current was found with different values for the same area size of graphene on different FTPs shown in Fig. S8. Here we demonstrated graphene transfer onto the unmodified-FTPs, found output voltage was 0.008 V for graphene transferred onto the thin CYTOP, TOPAS and PET (Fig. S8a–c), while graphene on surface engineered FTPs such as thicker PET (Fig. S8d) and higher Tg value polymers such as PI (Fig. S8e) output voltage was found to be increased due to wavy surface morphology of graphene. CVD graphene surface roughness increases the triboelectric effect^47^. Furthermore, it is observed that the electric signal also can be generated by hand driving periodically; the output result of device FTP/EVA/graphene/CYTOP VS ITO/PET was shown in Fig. 6c. And it was proved that the electricity generated by our device is effective when a 10 μF capacitor is charged successfully from 0 V to 3 V in less than 20 min, as Fig. 6d presented. Meanwhile, we also proved the electricity generated by our device is powerful enough to power LEDs, as 6 LEDs can be powered successfully as showed in Fig. 6e,f. This demonstration showed that the binding energy between graphene/EVA and the FTPs are strong enough and as well materials could be used as flexible and transparent electrode in the modern electronics. Even though only five polymer examples are shown, the procedure worked consistently on all other target substrates such as PC and PVC which justifies that our graphene transfer is universal.

## Conclusion

We have demonstrated a stamping method to transfer graphene onto different FTPs using EVA as binder only between graphene and target substrates without affecting the Cu substrates which could be used for repeated graphene growth. Surface modification of FTPs to alter the effective surface interaction with the EVA widens the choice for target substrates. Our transfer method is simple and fast, which ensures the clean and efficient transfer without inducing any damage either onto the graphene/substrates or Cu foil. What’s more, the electrical output performance is demonstrated with the fabrication of TENG and implied that our transfer method is realistic to scale up the graphene on plastics for industrial-scale. We believe this approach may be further improved by adopting effective strategies like metal nanowire based graphene transparent and flexible electrode reported elsewhere to replace ITO in optoelectronic devices^24^.

## Supplementary information

Supplimentary - Revised
