# Supplementary material for: A Universal Stamping Method of Graphene Transfer for Conducting Flexible and Transparent Polymers
Source: Sci Rep. 2019 Mar 8;9:3999. doi: 10.1038/s41598-019-40408-w (PMC6408549; doi:10.1038/s41598-019-40408-w)
Supplement: Supplementary file 1 — Supplimentary - Revised [file 41598_2019_40408_MOESM1_ESM.pdf]

Supplementary Information

A Universal Stamping Method of Graphene Transfer for Conducting Flexible and Transparent Polymers

Bananakere Nanjegowda Chandrashekar[+1], Ankanahalli Shankaregowda Smitha[+1,2], Yingchun Wu[3], Nianduo Cai[1], Yunlong Li[1], Ziyu Huang[3], Weijun Wang [1], Run Shi[1,4], Jingwei Wang[1,4], Shiyuan Liu[1], S. Krishnaveni[5], Fei Wang*[3], Chun Cheng*[1]

[1]Department of Materials Science and Engineering and Shenzhen Key Laboratory of Nanoimprint Technology, Southern University of Science and Technology, Shenzhen 518055, P. R. China

[2]Department of Electronics, Yuvaraja's College, University of Mysore, Mysuru-570006, India.

[3] Department of Electrical Engineering, Southern University of Science and Technology, Shenzhen 518055, P. R. China

[4]Department of Physics and Centre for 1D/2D Quantum Materials, The Hong Kong University of Science and Technology, Clear Water Bay, Kowloon, Hong Kong, P. R. China

[5]Department of Studies in Physics, University of Mysore, Mysuru, India

* Correspondence and requests for materials should be addressed to

Fei Wang (wangf@sustc.edu.cn) and Chun Cheng (chengc@sustc.edu.cn)

1. **Stamping process:**

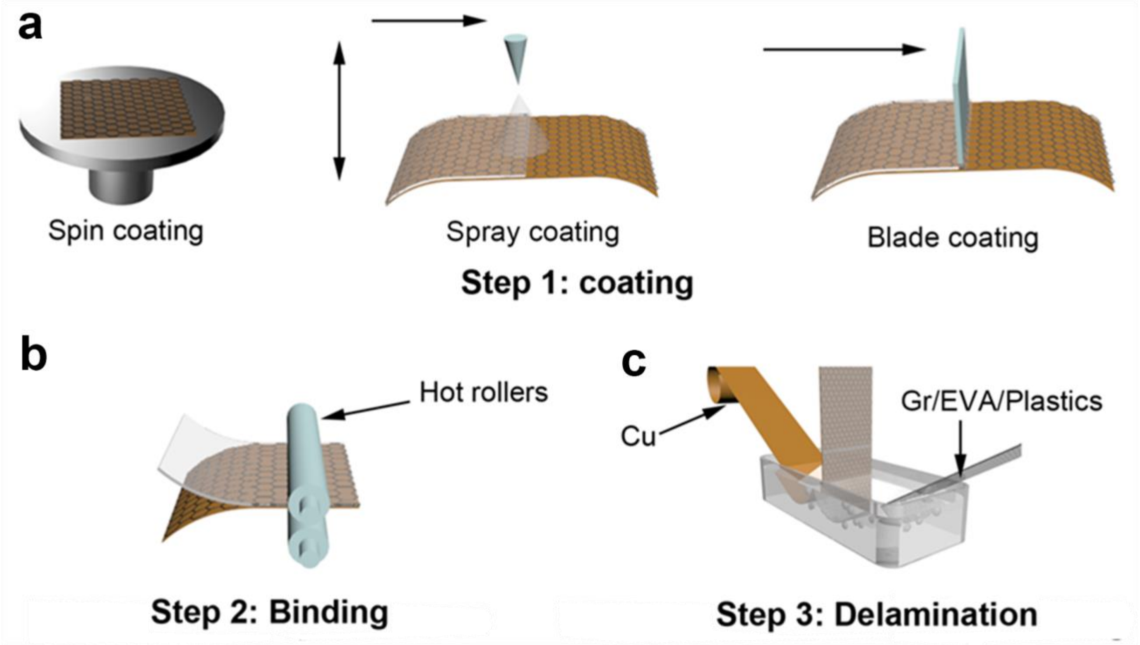

Figure S1: Schematic illustration of detailed stamping steps: **(a)** Step 1: Coating accomplished with three types; Spin coating limited to the small area of graphene while spray and blade coating applied for large area of graphene. Arrow mark represents the direction of the coating process. **(b)** Step 2: EVA/graphene/Cu binding with FTPs by passing EVA/graphene/Cu and plastics between two rollers, roller temperature were set at 120-140°C. **(c)** Step 3: Hydrogen bubbling method showing the delamination of graphene onto the target plastic substrates, the FTP/EVA/graphene/Cu was cathodically polarized at 2V, and H_2 bubbles were generated between graphene and Cu which facilitates the graphene to strip off from Cu onto the FTPs.

2. **Quality evaluation of CVD grown graphene on Cu transferred onto SiO2 by PMMA mediated wet transfer method.**

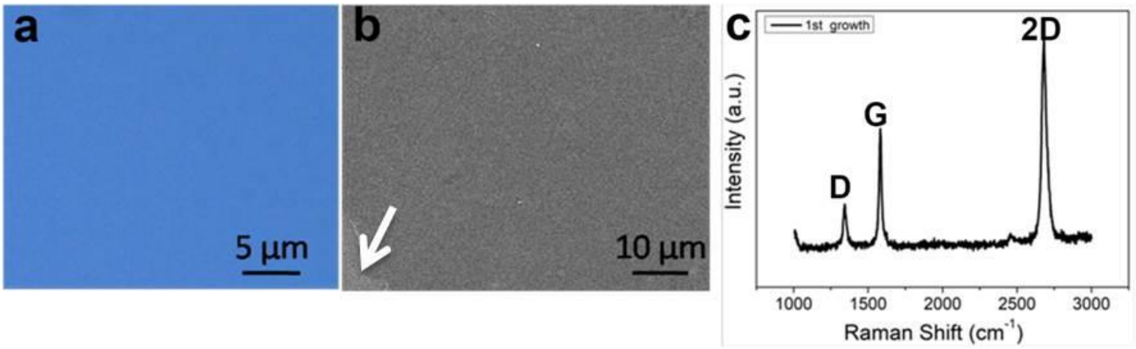

Figure S2: Quality Evaluation of CVD grown graphene: (**a**) Optical microscopy (OM) image of graphene transferred onto SiO_2/Si via PMMA mediated chemical etching method showing continuous graphene. (**b**) SEM image of graphene showing continuity without any pinholes while white arrow represents the background of SiO_2/Si. (**c**) Raman spectra of graphene transferred onto SiO_2/Si confirms the monolayer graphene with the ratio of 2D/G peak is more than 1

3. EVA thin film coating onto graphene/Copper

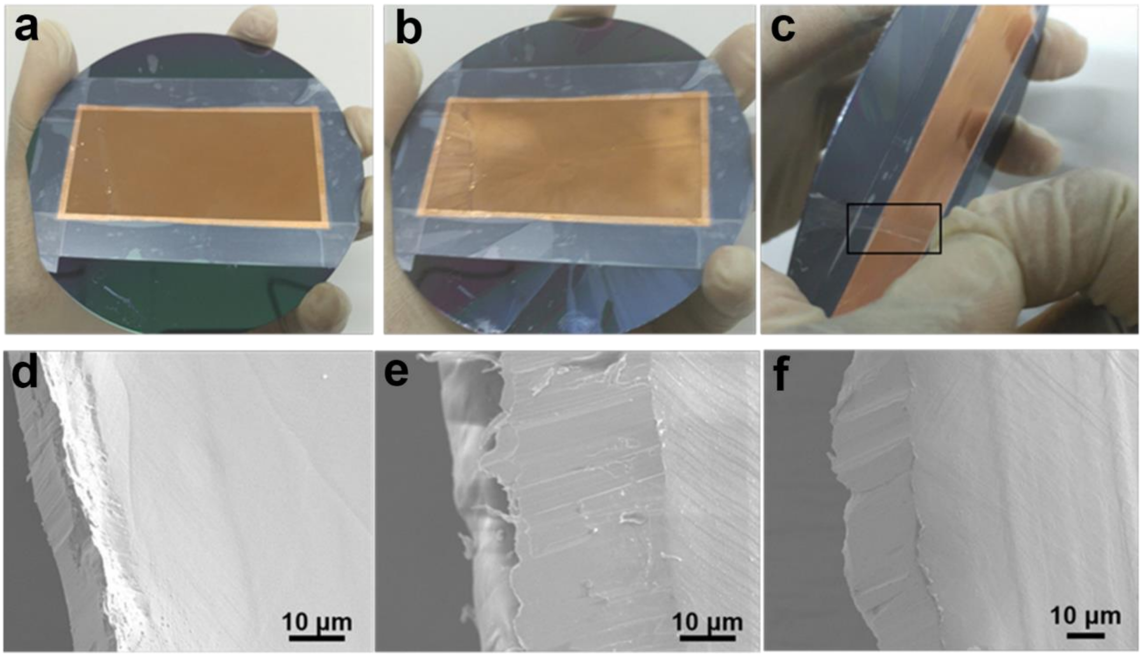

Figure S3: EVA coating process on graphene/Cu and its surface morphology: Photographs of **(a)** graphene/Cu were fixed onto flat surface before coating; All sides of graphene/Cu were covered with scotch tape to ensure the flat thin film and avoid the polymer residue remained backside. **(b)** graphene/Cu after EVA coating to form EVA/graphene/Cu. **(c)** Peeling off (black square) the coated EVA film on Cu substrates confirms that the deposition process ensures the continuous film. **(d)** Cross sectional SEM images of graphene/Cu before EVA coating showing the grain boundary, graphene/Cu after EVAcoating **(e)** shows thin continuous film covered the Cu morphology and Cu after EVA/graphene **(f)** transferred onto FTPs showing no EVA residue remains and mimics the Cu morphology before coating, which evidenced that the stamping method doesn't affects the Cu morphology.

4. Surface Engineering of more than 25µm thick polymers and higher glass transition temperature to that of EVA:

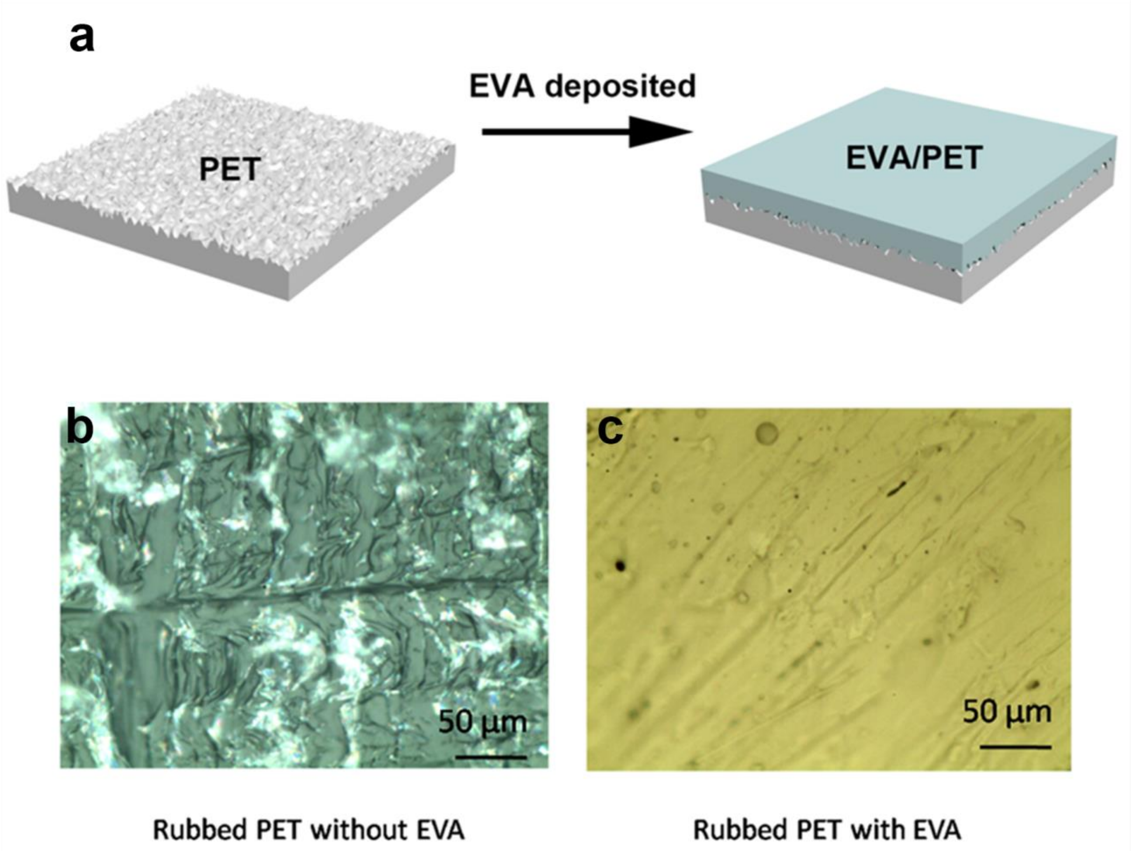

Figure S4. Surface engineering of polymer: (a) Schematic illustration of FTPs surface modification using sand blasting process Process also similar to others polymers (less than 25µm thickness) either smooth surface or higher/lower Tg value. Left, shows the surface roughness of a plastic, which is attributed to the tight clamping for the binding agent EVA. Right, shows that EVA coating onto the rough plastic gives smooth surface, which makes mechanically robustness an important factor for graphene transfer. OM of **(b)** 150 µm thicker PET plastic rubbed with sand paper makes the surface rough by crest and trough, which helps to bind EVA more tightly. **(c)** Crest and trough of rubbed PET was coated with EVA by spin coating showing the uniform surface.

5. AFM characterization of surface engineered polymer substrates:

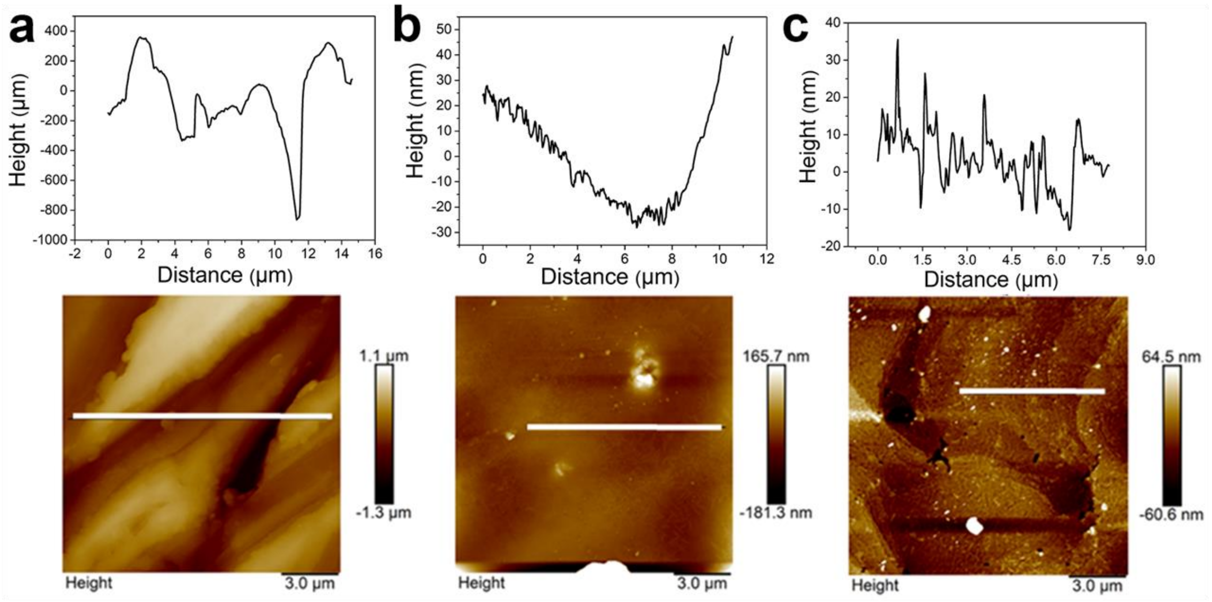

Figure S5: AFM topographic images to show the height profile of PI rubbed with sandpaper **(a)** where white line represents the line section to show that the width of crest and trough (µm). **(b)** EVA coated onto the rubbed PI and line section along the white line shows that crest and trough (nm) width is decreased upon EVA coating. **(c)** Crest and trough width were found to be increased upon graphene transferred onto the EVA coated on rubbed PI plastics. In the hot lamination, both Cu and surface engineered FTPs morphology induced on to the FTPs/EVA/graphene, shown in the white line along (c).

6. Graphene grain morphology effect on sheet resistance:

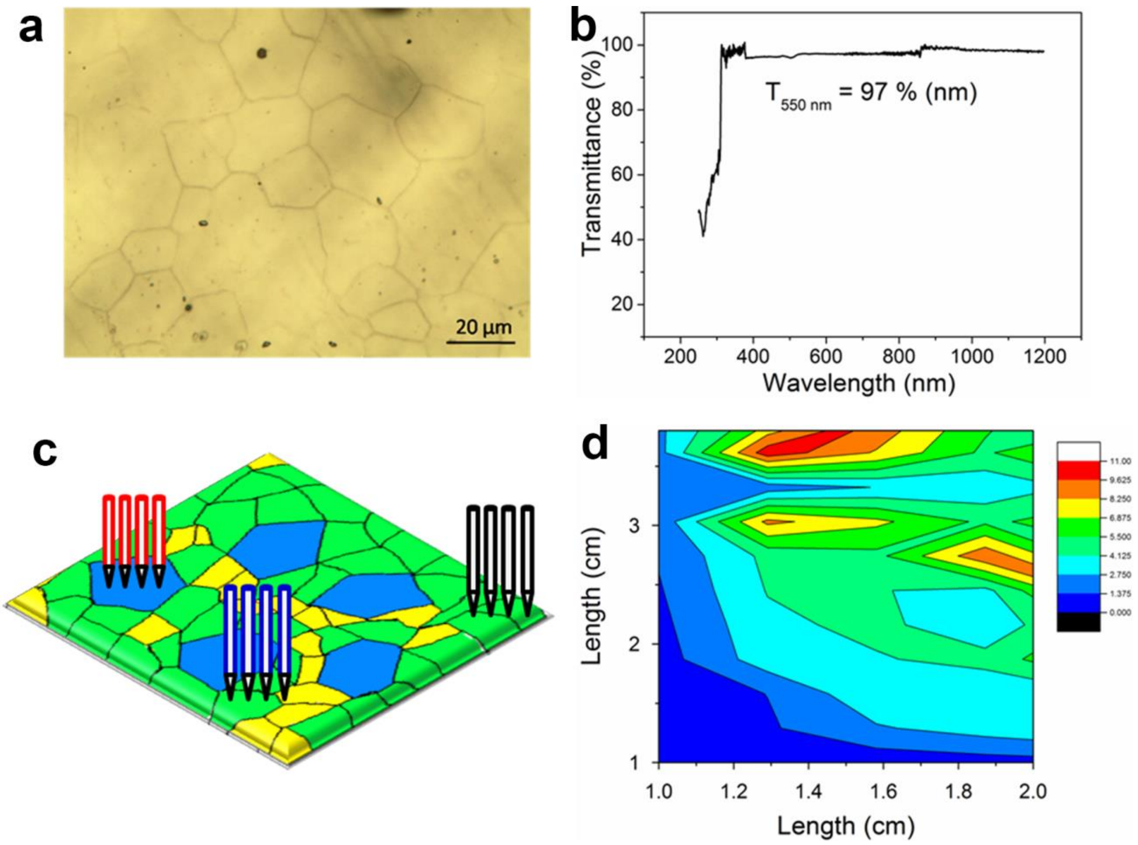

Figure S6: Characterization of graphene transferred onto FTPs by stamping method: (a) OM image of as grown graphene on electropolished Cu foil transferred onto FTPs by stamping method showing that the CVD graphene is polycrystalline, which makes Rs value higher. **(b)** UV-Visible spectrum of graphene transferred onto to the FTPs. Note that that EVA/FTPs absorption was subtracted, which results only graphene transmittance. **(c)** Schematic illustration showing grain size and its boundary effects the variation in sheet resistance measurement. Four probes spotted on different grain size of graphene crystals, for instance probes spotted (red color) away from the grain boundaries shows less sheet resistance as compared to the probes spotted near to the grain boundaries (black colour). **(d)** Distribution plot of sheet resistance of graphene/EVA/FTPs showing 1 to 11 KΩ/□.

7. Triboelectric Nanogenerator working frequency.

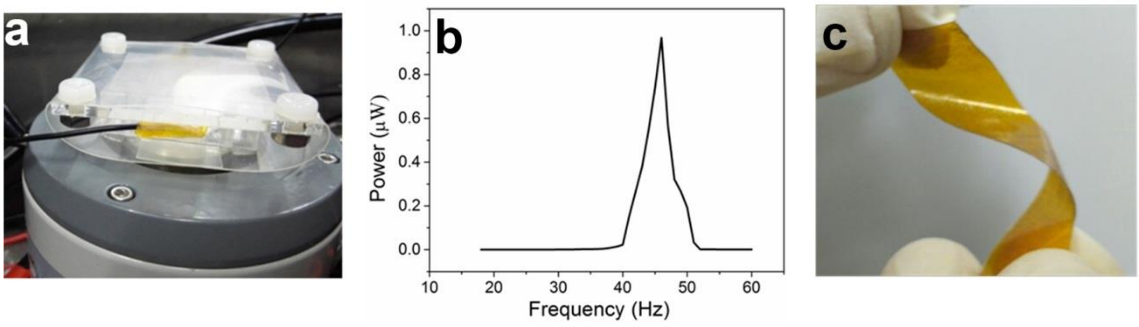

Figure S7: (a) Photograph of vibration test setting for triboelectric device. **(b)** Frequency scan test for device to explore the optimal work frequency. **(c)** Photograph of the graphene EVA/PI used to fabricate the triboelectric nanogenerator, which shows that the graphene/EVA clamped very tightly after being twisted.

8. Output voltage from graphene on unmodified and surface engineered FTPs

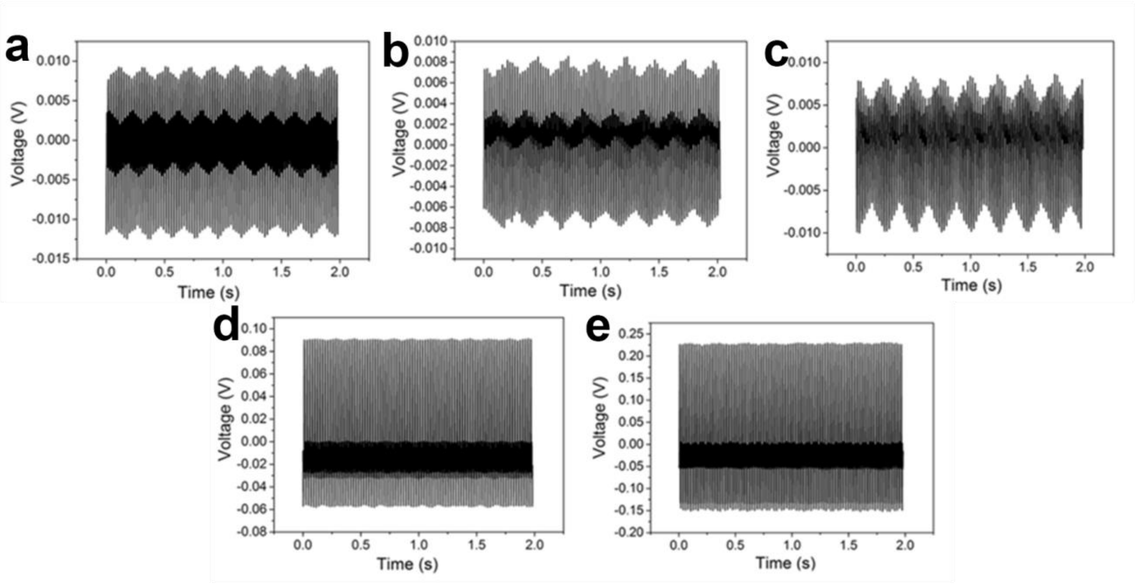

Figure S8: Electrical output performance of triboelectric nanogenerator: Top row, EVA/graphene on unmodified- FTPs such as PET **(a)**, Topas **(b)** and cytop **(c)**. Bottom row, graphene/EVA on surface engineered - FTPs such as PET **(d)** and PI**(e)**.
